# Clay minerals as a source of cadmium to estuaries

**DOI:** 10.1038/s41598-020-67279-w

**Published:** 2020-06-26

**Authors:** Weiduo Hao, Teruhiko Kashiwabara, Rong Jin, Yoshio Takahashi, Murray Gingras, Daniel S. Alessi, Kurt O. Konhauser

**Affiliations:** 1 Department of Earth & Atmospheric Sciences, University of Alberta, Edmonton, Alberta T6G 2E3 Canada; 2 Japan Agency for Marine–Earth Science and Technology (JAMSTEC), 2-15, Natsushimacho, Yokosuka, Kanagawa 2370061 Japan; 3 Department of Earth and Planetary Science, The University of Tokyo, 7-3-1 Hongo, Bunkyo-ku Tokyo, 1130033 Japan

**Keywords:** Environmental sciences, Environmental chemistry, Geochemistry

## Abstract

Given the high surface reactivity of clay minerals, it is assumed that flocculation will lead to metal accumulation in marginal marine settings. However, the degree of metal sorption to clays is impacted by solution pH and ionic strength, and it remains unknown whether riverine clays indeed serve as a metal sink once they encounter seawater where pH and ionic strength markedly increase. Here, we conducted cadmium (Cd) adsorption experiments to three types of common clay minerals – kaolinite, illite and montmorillonite. We found that 20–30% of Cd from illite and montmorillonite surfaces were desorbed when transitioning from freshwater to seawater pH and ionic strength conditions, while kaolinite showed no discernible differences. Synchrotron X-ray adsorption spectroscopy confirmed that Cd release corresponded to a change in bonding from outer- to inner-sphere complexes when clays encountered seawater pH and ionic strength conditions. If other trace nutrients (such as Cu, Zn, Co) adsorbed onto riverine clay minerals behave in a similar manner to Cd, we speculate that their desorption in marginal marine settings should exert a significant impact on the productivity of the biosphere.

## Introduction

Clay minerals are characterized by their high chemical reactivity^1–4^, and as major components in the suspended sediments of rivers^5–8^, they demonstrate a considerable influence on the transfer of trace elements from land to the oceans^9,10^. Differences between the chemistry of rivers (pH ranging from 2 to 8, ionic strength <0.01 M) and oceans (average pH 8.1, ionic strength from 0.56 M to 0.7 M) result in variations in both the aqueous speciation of trace elements and clay surface properties. This, in turn, affects the capacity with which metals stay bound to clays. For instance, previous studies indicated that increases in solution pH promote the adsorption of trace elements (e.g., Cu, Zn, Co and Cd) onto clay minerals, while conversely, high ionic strength (hereafter abbreviated as IS) inhibits their adsorption^11–13^. These phenomena are explained by the fact that increased pH enhances the deprotonation of surface functional groups, while high ionic strength attenuates the negative electrostatic field, leading to diminished cation adsorption at the clay surface^14,15^. Understanding how changes in aqueous chemistry during the transition from riverine to marine settings (e.g., within estuaries) impact trace metal sorption behaviour on clay surfaces is critical to determining the degree to which they either serve as a sink for metals to the bottom sediments or a source of metals to the water column.

The interactions between Cd and clay minerals is well studied due to its relatively simple aqueous speciation and potential as an environmental pollutant^16–20^. Cadmium is usually found as a divalent cation in environmental settings and it can also form complexes with organic matter and inorganic anions^21^. Previous studies have suggested that clay minerals are a significant sink for Cd under seawater conditions because of elevated pH versus freshwater environments^22^. However, the impact of increased IS on clay-Cd interactions could be more significant than the change in pH, especially the attenuation of the electrostatic field induced by increasing solution ionic strength^15^ (field data see Supplementary Table 1). The adsorption/desorption behaviour of trace elements, including Cd, on clays as they transit from rivers to estuaries is still poorly resolved, despite potentially critical impacts on the near-shore biosphere.

The type of chemical binding determines the stability of adsorbed species and regulates the remobilization of the metal from clay surfaces when environmental conditions change^23–38^. Grafe *et al*. (2007) and Du *et al*. (2016) revealed that Cd binding onto kaolinite and montmorillonite forms an outer-sphere complex coordinated by 6 O (oxygen) at around 2.2 Å from Cd^29,34^. Similarly, Saijdu *et al*. (2008) studied Cd adsorption onto natural mixed clay minerals, and found that Cd is primarily bound as an outer-sphere complex which is octahedrally-coordinated with O^31^. Takamatsu *et al*. (2006) analysed the local structure of the Cd-montmorillonite complex and found that Cd formed outer-sphere complexes in low pH environments, but formed surface precipitates at pH higher than 7.1^33^. By contrast, Vasconcelos *et al*. (2008) showed that Cd formed inner-sphere complexes with Al or Si on clay surfaces at high pH^32^. Despite literature on the local structure of Cd adsorption on clay minerals, knowledge on the role of clays in the transport of Cd from rivers to the ocean and variations in its adsorption coordination environment during this transition remains absent. As such, in this study we have addressed the question of Cd adsorption behaviour onto clay surfaces and the corresponding local structure variations between freshwater and seawater pH and IS conditions.

In this work, we tested variations in Cd affinity toward three common clay minerals, kaolinite, illite and montmorillonite, as a function of increasing pH and ionic strength to determine whether clay minerals release or sequester Cd at marginal marine pH and IS conditions. We purposefully did not consider the full complexity of seawater per se (e.g., the effects of co-ions or dissolved organic matter) because to ascertain the effects of pH vs. ionic strength necessitated that we simplify the composition of our aqueous solution. Surface complexation modelling (SCM), informed by Extended X-ray Adsorption Fine Structure (EXAFS) analysis, was performed on Cd-bearing-clay samples to better understand the binding environment of Cd at freshwater and marine aqueous conditions.

## Materials, experiments and methods

### Clay minerals preparation

Samples of kaolinite (KGa-2), montmorillonite (SWy-2), and illite (IMt-2) were obtained from the Clay Mineral Society, Source Clays Repository (Purdue University, West Lafayette, USA). All clays were grounded to pass a 100-mesh sieve and washed by 0.01 M HNO_3_ to remove impurities. They were then washed three times by suspending 0.5 g of clay in 50 mL of 0.1 M sodium nitrate solution (ACS certified, Fisher Scientific) for 3 h and then centrifuged at 10,000 g for 20 min. After washing, clays were frozen to −20 °C for 12 h and then freeze dried^14^.

### Adsorption isotherms

Cadmium (Cd) was adsorbed onto the three clay minerals at different initial Cd concentrations (1, 5, 10, 20, 40, 60, and 80 ppm) in order to construct Cd sorption isotherms. A 1000 ppm CdCl_2_ stock solution was made by dissolving CdCl_2_ salt (ACS certified, Fisher Scientific), after which Cd solutions of the above concentrations were prepared by diluting the 1000 ppm solution into polypropylene tubes to a total volume of 50 mL. A 0.01 M NaCl solution was used during dilutions to buffer solution ionic strength. Inductively coupled plasma – mass spectrometry (ICPMS; Agilent 8800) was used to precisely determine the initial Cd concentrations in these solutions. Finally, 45 mg of clay was added to each solution to make a 1 g/L solid suspension. After agitation, the pH of each solution was adjusted to 6 by adding small aliquots of 0.1 M HCl and 0.1 M NaOH. Another set of adsorption isotherms using the same Cd concentration was performed at pH = 8 and ionic strength = 0.56 M (NaCl salt was used to adjust ionic strength). The solution pH was monitored and repeatedly adjusted to maintain the solution pH during the adsorption period (24 h). Once equilibrium was reached, 5 mL of clay suspension was pipetted and filtered using a 0.2 µm PTFE filter. The filtrates were acidified by 2% HNO_3_ and 0.5% HCl solution for ICP-MS analysis.

### Desorption experiments under simulated estuary conditions

To ascertain Cd behavior on clay surfaces when aqueous pH and IS conditions change from freshwater to seawater, laboratory experiments were performed to simulate this transition between aqueous conditions. The initial freshwater pH and IS adsorption experiment was equilibrated for 24 h. Following this, the clay was adjusted to seawater pH and IS conditions, and the resulting solution was sampled over 3 days, with time intervals (at 0 h, 1 h, 2 h, 4 h, 8 h, 12 h, 1 d, 2 d, 3 d) while the solution was maintained at pH 8. Specifically, a 0.01 M NaCl solution was added into a beaker, after which CdCl_2_ stock solution was added to produce a 1 ppm Cd solution. A magnetic stir bar kept the solution mixing at 240 rpm during the experimental period. During the experiments the reaction beaker was covered by Parafilm to prevent possible solution evaporation. Once well mixed, a 5 mL aliquot was taken and filtered for initial Cd concentration analysis. After, the solution pH was adjusted to 6 by adding small aliquots of 0.1 M HCl and 0.1 M NaOH to mimic freshwater pH and IS conditions, 0.395 g of the target clay mineral was then added into the beaker to make a 1 g/L clay suspension. The adsorption of Cd onto clays at freshwater pH and IS conditions lasted 24 hours and the pH was maintained at 6 during this period by acid-base adjustment.

After 24 h, a final aliquot was taken and filtered through a 0.2 µm PTFE to determine the degree of Cd adsorption at freshwater pH and IS conditions. The solution pH was then adjusted to 8 using aliquots of a 0.5 M NaOH solution, while 12.87 g of NaCl salt was added into the beaker to increase the IS to 0.56 M; these pH and ionic strength conditions were used to represent seawater-like conditions. Once the pH was stable, a 5 mL aliquot was sampled and filtered for Cd concentration analysis representing Cd adsorption at t = 0 h. The same procedure was applied for all of the subsequent samples in the time series. All collected samples were acidified by 0.5% HCl and 2% HNO_3_ prior to Cd analysis by ICP-MS.

### pH edge experiments

Cadmium pH adsorption edge experiments were performed to compare how pH impacts Cd adsorption behavior under freshwater and seawater IS conditions. Initially 100 mL of 0.56 M NaCl was added to a glass beaker and a solution Cd concentration of 1 ppm was achieved by adding CdCl_2_ stock solution. The resulting solution was stirred for 5 minutes before a 10 mL initial sample was taken. The target clay was then added to the Cd solution to create a 1 g/L slurry. During continuous agitation, 10 mL aliquots of the slurry were transferred to seven 15 mL polypropylene test tubes. Following transfer, the individual test tubes were pH adjusted using small aliquots of 1 M, 0.1 M, and 0.01 M HCl and NaOH across a pH range of 3 to 9. Freshwater IS condition pH edge experiments were performed in the same way, but the IS was instead buffered by a 0.01 M NaCl solution.

### Surface complexation modelling

Surface complexation modelling of Cd adsorption pH edge data was performed using the software FITEQL (4.0)^39^. Surface protonation equations and corresponding constants and site concentrations were utilized from previous studies^14,15^. The system of equations to represent Cd adsorption are:

R1 $$\equiv {\rm{LH}}+{{\rm{Cd}}}^{2+}\leftrightarrow \equiv {{\rm{LCd}}}^{+}+{{\rm{H}}}^{+}$$

1 $${{\rm{K}}}_{{\rm{LCd}}}=\frac{[\,\equiv \,{{\rm{LCd}}}^{+}]\cdot {\alpha }_{{{\rm{H}}}^{+}}}{[\,\equiv \,{\rm{LH}}]\cdot {\alpha }_{{{\rm{Cd}}}^{2+}}}$$

R2 $$\equiv {\rm{XOH}}+{{\rm{Cd}}}^{2+}\,\leftrightarrow \,\equiv {{\rm{XOCd}}}^{+}+{{\rm{H}}}^{+}$$

2 $${{\rm{K}}}_{{\rm{XOCd}}}=\frac{[\,\equiv \,{{\rm{XOCd}}}^{+}]\cdot {\alpha }_{{{\rm{H}}}^{+}}}{[\,\equiv \,{\rm{XOH}}]\cdot {\alpha }_{{{\rm{Cd}}}^{2+}}}$$

Surface functional groups for the clays were assigned to one of two types: ≡LH or ≡XOH, which represent a permanently charged (i.e., structural) surface functional group (one that can only deprotonate) and an amphoteric surface functional group (representing Si-OH and Al-OH groups), respectively^13,40,41^. Both non-electrostatic (NEM) and constant capacitance models (CCM) were applied to fit the experimental results. The specific modelling procedure and surface acidity constants of protonation-deprotonation reactions were taken from our previous study^15^. A Na^+^ exchange reaction was applied to the ≡LH sites of all three clay minerals because the clay’s interlayer has an inherently high ion exchange capacity. The proton interaction constants of the IS = 0.01 M condition and 0.56 M condition were extrapolated from our previous research^15^. Aqueous complexation constants for the hydrolysis of Cd(II) and chloride complexation with Cd(II) were taken from Baes and Mesmer^42^ and activity coefficients calculated using either the Davies equation (seawater conditions) or the Debye-Hückel equation (freshwater conditions) because of the difference in IS between freshwater and seawater conditions^22^. The detailed input parameters are listed in Supplementary Table 2.

### Synchrotron X-ray adsorption spectroscopy

The coordination environment of Cd adsorbed onto the three clay minerals was determined by synchrotron Extended X-ray Adsorption Fine Structure (EXAFS) spectroscopy. Samples were prepared by performing Cd adsorption (1 ppm) onto kaolinite, illite, and montmorillonite at freshwater and seawater pH and IS conditions, respectively. Cadmium K-edge EXAFS spectra were measured at beamline BL01B1 of SPring-8 (Hyogo, Japan). The white beam from a bending magnet was monochromatized by a Si(311) double crystal monochromator. Two Rh coated mirrors, placed above and downstream of the monochromator, were used for collimation and focusing of the X-ray beam. Harmonic rejection was achieved by adjusting the glancing angle of these two mirrors. The beam size was adjusted by slits to *ca*. 1 mm (V) × 6 mm (H) on the sample placed at 45° to the incident X-ray^37^. Intensities of incident and transmission X-rays were monitored with ionization chambers up and downstream of the samples, and fluorescence X-rays were monitored with a 19-element Ge sold-state detector placed at 90° to the incident X-ray. The Cd K-edge EXAFS spectra for all the adsorbed clay samples were measured in fluorescence mode. The X-ray energy was calibrated with the first peak of CdO at 26.7159 keV.

EXAFS analyses were performed using REX2000 ver. 2.5 (Rigaku Co.). The background was removed from the raw spectra by a spline smoothing method, and *E*_0_ was set at the edge inflection point for all of the studied samples. The χ(k) functions were extracted from the raw spectrum by a spline smoothing method. The Fourier transformation was performed to convert *k*^3^χ(*k*) oscillations from *k* space to *r* space in the range from 3.25 to 7.70 Å for all the samples. The inversely Fourier filtered *k*^3^χ(k) spectra were analyzed with a curve-fitting method, where the theoretical backscattering amplitudes and phase-shift functions for Cd-O, Cd-Cl, Cd-Si, and Cd-Al were calculated from the structure of Cd(OH)_2_, CdCl_2_, and Cd_4_(Al_8_Si_8_O_32_) (H_2_O)_17.35_ using FEFF 8.5^43–45^.

## Results and discussion

### Cd behavior on clay surfaces from freshwater to seawater conditions

**Figure 1:**
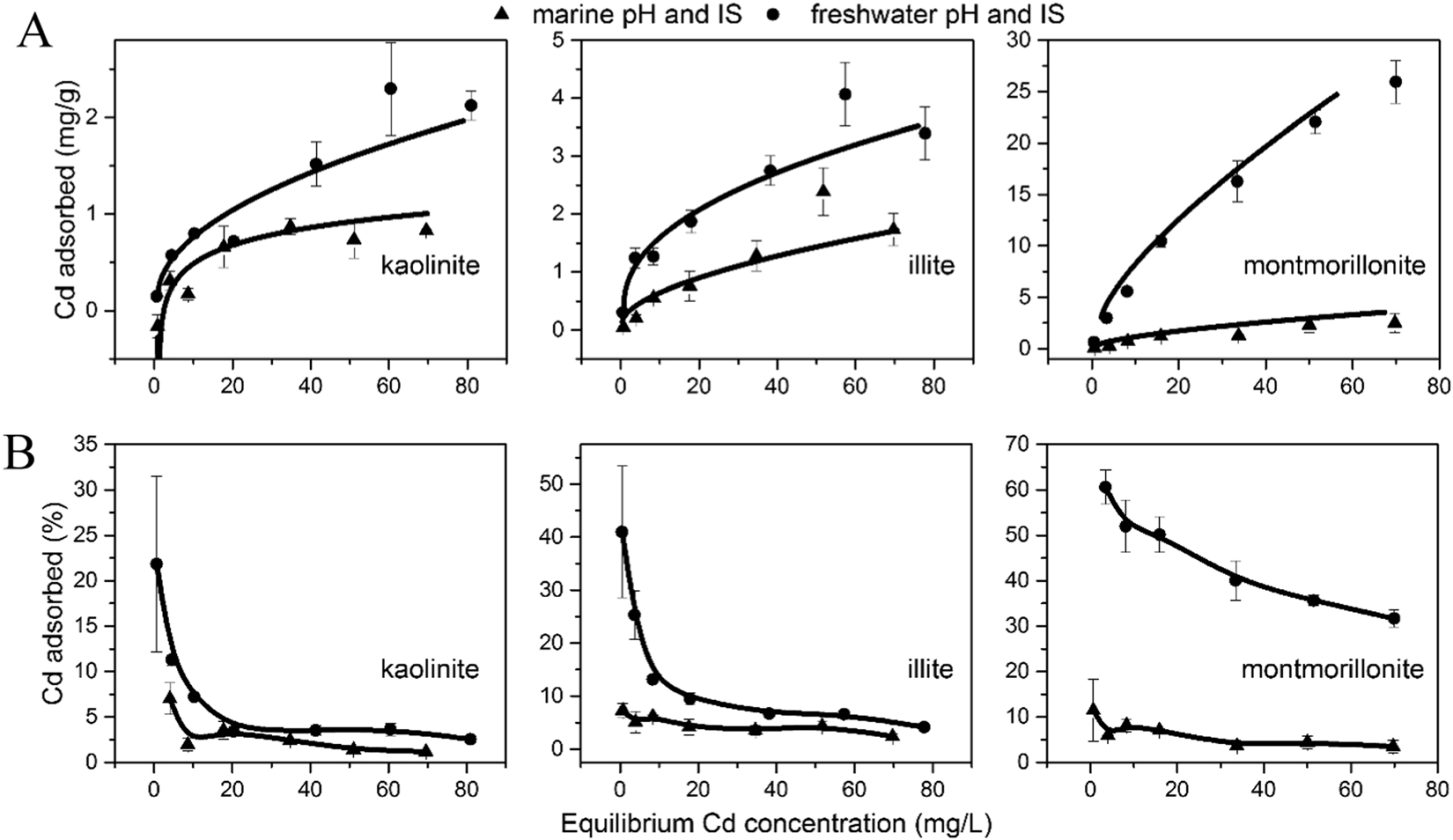
Comparison of Cd adsorption onto three tested clay minerals at freshwater (filled circle) and marine (filled triangle) pH and IS conditions. Filled circles are experimental adsorption data; curves are Langmuir model fits. Equilibrium was reached after 24 h. Error bars represent the standard deviation of duplicates. (**A**) the Cd adsorption is in units of mg/g; (**B**) the Cd adsorption is in units of %. (Figure is generated by Origin 9.1, https://www.originlab.com/).

The Cd adsorption isotherms show that the freshwater pH and IS conditions have a higher degree of Cd binding than the seawater counterparts (Fig. 1). At the maximum Cd concentrations used (80 ppm), kaolinite shows the smallest difference between freshwater and seawater conditions, with only 1.5 mg/g difference. Illite showed a 2 mg/g difference, while montmorillonite displayed the largest difference with more than 20 mg/g.

A Langmuir adsorption isotherm was applied to derive the maximum Cd adsorption capacity (Supplementary Table 4) for three clay minerals by using the following equation:

3 $$\frac{{C}_{e}}{{q}_{e}}=\frac{1}{{q}_{m}{K}_{L}}+\frac{{C}_{e}}{{q}_{m}}$$

where, C_e_ is the equilibrium concentration of Cd (mg/L), q_e_ is the amount of solute adsorbed per unit weight of sorbent (mg/g), q_m_ is the Langmuir constant which represents the saturated monolayer sorption capacity (mg/g), and K_L_ is the Langmuir constant. The maximum Cd adsorption capacity experienced a decreasing trend from freshwater to seawater pH and IS conditions (Supplementary Table 4). This discrepancy between freshwater and seawater pH and IS conditions is particularly large for montmorillonite which decreased from 36.76 mg/g to 4.73 mg/g, while kaolinite and illite decreased from 2.31 mg/g to 1 mg/g, and 3.59 mg/g to 1.94 mg/g, respectively. In summary, the results of the Cd adsorption isotherms indicate that when clays are transported from riverine to marine conditions, the suppression caused by the increase in IS is more significant than the promotion caused by the increase in pH. This then relates to differences between the 3 clays as follows: Illite has relatively stronger surface electrostatic field as compared to montmorillonite^5^, which means it should have more Cd adsorption than montmorillonite; however, the swelling property of montmorillonite ensures a higher adsorption capacity compared to illite under freshwater pH and IS conditions. Then, when montmorillonite encounters seawater conditions, it flocculates, and the collapse of interlayer space leads to the release of a large portion of Cd from interlayer. We suggest that this may be the reason why Cd desorbed more significantly from montmorillonite surfaces once subjected to seawater conditions than from illite.

**Figure 2:**
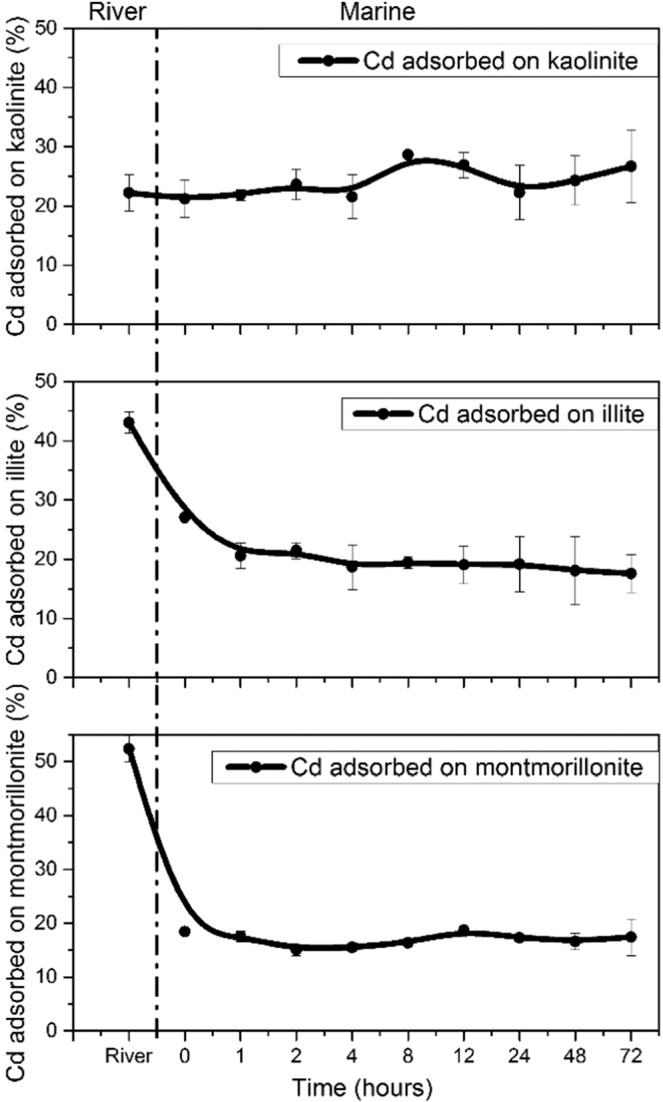
Adsorption of Cd onto three tested clay minerals as a function of changing aqueous conditions from freshwater to seawater pH and IS. The area left of the dashed line represents the amount of Cd adsorbed under freshwater pH and IS conditions; the area right of the dashed line represents Cd adsorbed at marine pH and IS condition as a function of time after changing to marine condition (i.e., pH increases to 8.0 and ionic strength increases to 0.56 M). Error bars represent the standard deviation of replicates. (Figure is generated by Origin 9.1, https://www.originlab.com/).

The measured differences in adsorption provide further insights into how adsorbed Cd will behave when it encounters an estuary (Fig. 2). Under freshwater conditions, the amount of Cd removed from solution onto the surfaces of montmorillonite and illite is between 40% to 50%. However, once the solution was changed to seawater pH and IS conditions, Cd adsorption immediately decreased to approximately 20%. In contrast, kaolinite shows minimal difference in Cd adsorption (i.e., 25–30% adsorption at both freshwater and seawater conditions). This pattern is likely a consequence of the offset effect on Cd adsorption with increases in both pH and solution ionic strength. By comparison to illite and montmorillonite, kaolinite has less structural isomorphic substitution (e.g., the replacement of Al^3+^ by Mg^2+^), and thus the effect of suppressing Cd adsorption by attenuation of surface electrostatic field is relatively weaker. This likely explains why kaolinite shows little difference in terms of Cd adsorption via an increase in IS and pH, but illite and montmorillonite surface reactivity to Cd is significantly inhibited. It is worth noting that in our experimental results, the release of Cd under seawater conditions is simply due to changes in clay surface reactivity because of variations in pH and IS (specifically more Na^+^). In reality, there will be competitive adsorption reactions (such as Pb, Ni, and Cu), dissolved organic matter (DOC) chelation and changes in redox conditions (such as the precipitation of CdS due to sulphate reduction) that could also influence Cd behaviour^46^. As such, it is important to note that under seawater conditions, the release of Cd from clay surfaces could be more complex due to various aqueous conditions.

### Surface complexation modelling

**Figure 3:**
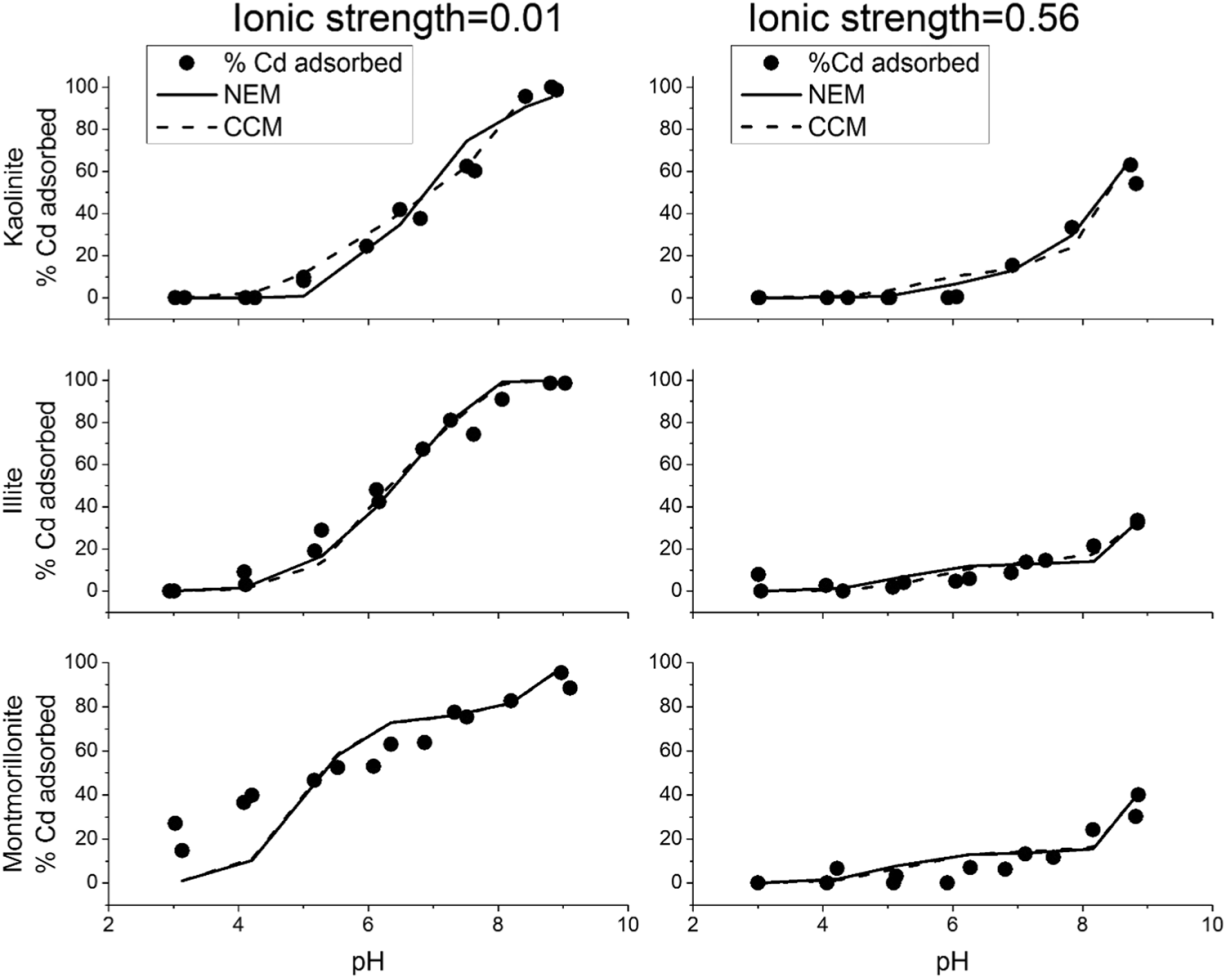
Cd pH adsorption edges and the fitting of experimental data by non-electrostatic and constant capacitance surface complexation models. The Cd initial concentration is 1 ppm. (Figure is generated by Origin 9.1, https://www.originlab.com/).

The degree of adsorption of Cd onto the tested clay minerals increased with solution pH (Fig. 3) as a result of increased deprotonation of functional groups and negative charge at the clay surfaces. Furthermore, the pH edge results show that Cd adsorption under seawater IS conditions is significantly depressed as compared to freshwater IS condition (Fig. 3). Kaolinite shows the least difference amongst the three studied clays in Cd adsorption under seawater IS (99% adsorption at pH = 9) compared to freshwater IS (70% adsorption at pH = 9). Cd adsorption onto illite is strongly suppressed at seawater IS, with only 32% adsorbed at seawater IS and pH = 9 compared to 98% adsorbed at freshwater IS (pH = 9). This behaviour is similar to montmorillonite, showing 95% adsorption at pH = 9, IS = 0.01 M and 40% adsorption at pH = 9, IS = 0.56 M.

NEM and CCM surface complexation models show little difference for the fitting of Cd adsorption data (Fig. 3), indicating that both models can successfully predict Cd adsorption behaviour. The modelled montmorillonite adsorption shows minimal deviation compared to experimental data, which may be attributed to the swelling property that can accommodate more Cd in the interlayer. The detailed modelling procedure, including input parameters and output results, is provided in Supplementary Table 3.

**Figure 4:**
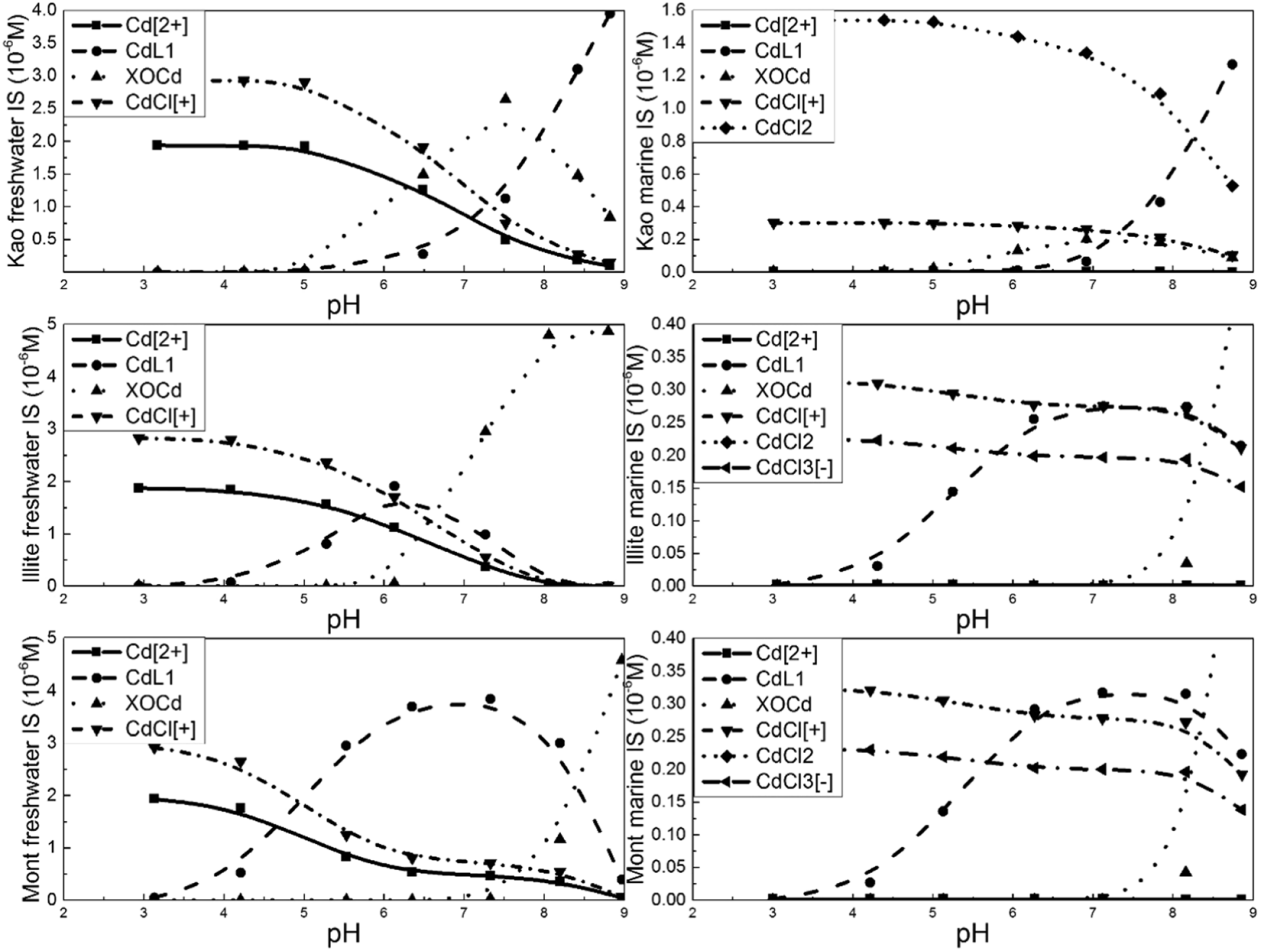
Cd speciation diagram as a function of pH under freshwater and marine IS condition. (Figure is generated by Origin 9.1, https://www.originlab.com/).

Cd speciation diagrams at freshwater and seawater IS were calculated based on the SCM results (Fig. 4). Compared to the freshwater IS condition, Cd was significantly mobilized by Cl under seawater IS condition, resulting in less Cd adsorption. This implies that Cl complexes are sufficiently strong to inhibit the adsorption of Cd onto clay surfaces, and it frees previously adsorbed Cd from clay surfaces. The surface species ≡XOCd^+^ is dominant for kaolinite under freshwater IS condition, while ≡LCd^+^ is the primary species under seawater IS conditions. By contrast, montmorillonite and illite both have ≡LCd^+^ as the predominant species at low pH, but this species concentration decreases as both pH and the concentration of ≡XOCd^+^ correspondingly increase. In summary, the collective results of modeling indicate Cd adsorption onto illite and montmorillonite is dominated by the ≡LH group under freshwater IS condition, while under seawater IS condition the ≡XOH group is the functional group most responsible for Cd adsorption. The distribution of Cd onto kaolinite surfaces shows the opposite trend.

### Coordination of Cd on clay mineral surfaces

**Figure 5:**
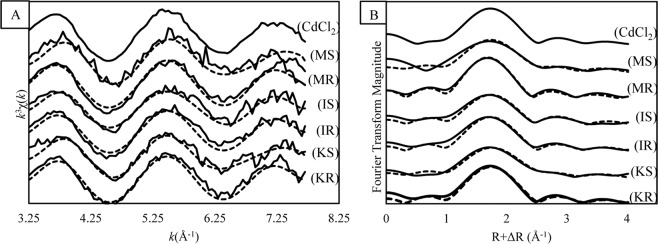
EXAFS analyses of Cd adsorption onto three clay minerals under freshwater and marine pH and IS conditions. (**A**) k3-weighted χ(k) spectra, (**B**) Fourier transformations of (**A**). The abbreviations represent: (KR) = kaolinite at river pH and IS conditions; (KS) = kaolinite at seawater pH and IS conditions; (IR) = illite at river pH and IS conditions; (IS) = illite at seawater pH and IS conditions; (MR) = montmorillonite at river pH and IS conditions; (MS) = montmorillonite at seawater pH and IS conditions; and (CdCl2) = CdCl2 aqueous solution. (Figure is generated by Excel, Microsoft office 365, https://www.office.com/).

Figure 5A,B show the fitting of *k*^3^-weighted χ(k) spectra of Cd K-edge EXAFS data and their Fourier transformations for a series of the samples, respectively. The *k*^3^-weighted spectrum of the montmorillonite sample at seawater pH and IS seems is noisy, which may be due to the low Cd concentration. By comparison, illite and kaolinite samples have relatively high Cd loading, which ensures a higher quality of spectrum compared to montmorillonite. We performed shell-by-shell fitting of the spectra by using several bonding pairs calculated by FEFF 8.5 including Cd-O, Cd-Cl, Cd-Si, and Cd-Al, and the Cd-O and Cd-Al bonds ended up better simulating the sample spectra. The spectra of Cd bound to the three clay minerals under freshwater pH and IS conditions are dominated by one Cd-O frequency with a coordination number (CN) around 6 at approximately 2.27 Å. This corresponds to a hydrated Cd ion surrounded by 6 water molecules, indicating that Cd dominantly forms an outer-sphere complex under freshwater aqueous conditions^34^. On the other hand, the first shells of the seawater samples in the R-space spectra seem to be broader compared to those of freshwater samples. This broadening of the first shell in R-space can be attributed to the formation of an inner-sphere complex that has a higher affinity to ≡AlOH functional groups at high ionic strength conditions in the seawater pH and IS samples: under seawater pH and IS conditions, a second shell of Cd-Al appears for all three clay minerals at R + ΔR = 3.16–3.36 Å, while it is absent for the samples at freshwater pH and IS. The Debye Waller factors (σ^2^) obtained by fixing the CN of Cd-O to 6 for all samples are systematically larger for seawater samples compared with freshwater samples (Supplementary Table 5), indicating thermal and static disorder of the Cd-O bonds in samples at seawater pH and IS conditions compared to freshwater conditions. We presume that this systematic increase in σ^2^ corroborates the mixture of several different Cd-O distances by sharing O with clay surface under seawater conditions as discussed in Kashiwabara *et al*.^38^. The differences in adsorption local structure between seawater and freshwater pH and IS conditions may be attributed to the collapse of clay electrostatic field due to the increase of IS, which decreases the amount of outer-sphere binding of Cd (no second shell). This then results in the inner-sphere form on Al adsorption sites (a second shell related to Al at the clay surface). In previous studies conducted by Takamatsu *et al*. (2006) and Vasconcelos *et al*. (2008), Cd inner-sphere complexes onto clay surfaces were observed at high pH compared to outer-sphere complex at low pH^32,33^, which corroborates our results of inner-sphere complexes preferentially existing under seawater pH and IS conditions.

Under seawater conditions, the CN of Cd on Al site is 1.5 for illite, with an interatomic distance of 3.35 Å. This indicates a bidentate mononuclear (edge sharing) complex coordination environment^32^. The Al-OH interatomic distance in Al-octahedron is around 2 Å, while Cd-O is 2.3 Å. Through bidentate mononuclear (edge sharing) complex, there is likely a distortion in the Cd octahedron leading to a longer interatomic distance around 3.35 Å. By contrast, kaolinite and montmorillonite have a CN around 2, and a distance around 3.1 Å. This corresponds to a tridentate binuclear (plane sharing) complex where the Cd octahedron shares three oxygens from two Al octahedrons. Each Al-octahedron provides apex oxygen with Cd and the third oxygen is shared between the two Al octahedrons. Our interatomic distance corresponds to previous studies of Cd-exchanged zeolites with a distance of approximately 3.1–3.3 Å^45,47^. At present it is unclear to us why illite shows bidentate mononuclear bonding environment compared to tridentate binuclear bonding on kaolinite and montmorillonite surfaces at seawater pH and IS conditions.

**Figure 6:**
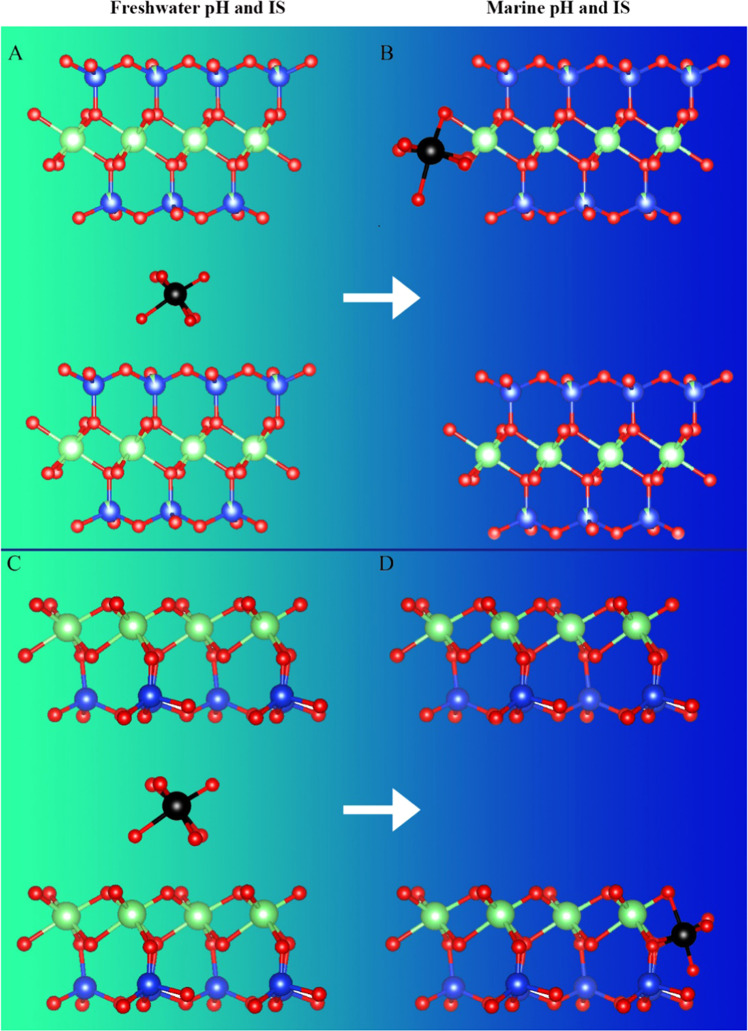
Molecular model of Cd adsorption onto clay minerals. (**A**) outer-sphere complexation of Cd onto 2:1 layer clays (e.g., Cd adsorption onto illite or montmorillonite under riverine pH and IS conditions); (**B**) inner-sphere complexation of Cd onto 2:1 layered clays (e.g., Cd adsorption onto illite or montmorillonite under marine pH and IS conditions); (**C**) outer-sphere complexation of Cd onto 1:1 layer clays (e.g., Cd adsorption onto kaolinite under riverine pH and IS conditions); and (**D**) inner-sphere complexation of Cd onto 1:1 layer clays (e.g., Cd adsorption onto kaolinite under marine pH and IS conditions). Black, red, blue and green balls represent Cd, O, Si, and Al, respectively. (Figure is generated by Adobe Photoshop CS 6, https://www.adobe.com/products/photoshopfamily.html).

A proposed molecular model of Cd adsorption is illustrated in Fig. 6. Edge site adsorption was observed for Cd binding onto clay surfaces under seawater pH and IS conditions based on the EXAFS results. Previous research shows that at high IS and pH conditions, trace elemental adsorption (e.g., Cu and U) tends to transfer from basal sites to edge sites^48,49^ due to their high energy and unsaturated bonding. This is a consequence of greater electrostatic attraction of cations at low IS conditions as compared to high IS where the electric field is attenuated^15^. At the same time, elevated pH in the seawater experiments promotes edge site deprotonation, which provides more Al sites for Cd adsorption. The preferential adsorption of Cd to Al sites may partially explain why kaolinite shows less Cd desorption from freshwater to seawater pH and IS conditions. Kaolinite is a 1:1 clay with Al-octahedral sheet exposed compared to illite and montmorillonite that the Al-octahedral sheet is sandwiched by two Si-tetrahedral sheets. Thus, Cd can form more inner-sphere complex with kaolinite compared to illite and montmorillonite. It is interesting that Cd-Cl complex adsorption was not detected in our experimental conditions even at the 0.56 M Cl^−^ concentration, indicating that Cd-Cl complexes are not strongly adsorbed to clays under seawater pH and IS conditions. This observation correlates to the higher Cd concentration in solution under seawater ionic strength, which is supported by the modeling results that Cd is mobilized by Cl^−^ under seawater conditions.

## Environmental implications

Our experimental results indicate that Cd can be released from clay surfaces in estuaries due to higher ionic strength, despite increased pH causing clay surface site deprotonation. Furthermore, these variations influence clay surface properties, thereby directly impacting the local binding environment of Cd at the various clay surfaces. For instance, we found that 20–30% of adsorbed Cd can be released as free cations (Cd^2+^) from illite and montmorillonite, while kaolinite shows little Cd release.

Our experimental findings correlate well with a compilation of Cd concentrations in suspended sediments of rivers worldwide (Supplementary Table 1)^50–63^ which shows that although the sampling season, methods and locations vary, there is a clear decreasing trend in Cd concentration in sediments from rivers to the ocean (an exception for the Seine River which has higher Cd concentration on suspended sediments within the estuary). The collective results corroborate what we observe in the experimental study, that being Cd is liberated from clays as they reach the intermediate pH and IS of estuaries and the seawater pH and IS open ocean.

The potential release of Cd from suspended clay minerals during this environmental transition could be significant because the annual discharge of suspended particles to the oceans is around 15 Gt per year^7^, with the <2 µm size fraction accounting for an estimated 10–50 weight % of those suspended particles^64,65^: in some rivers clay minerals contribute more than 90% of the <2 µm size fraction^10^. In terms of clay mineral composition in river suspended sediments, this varies substantially depending on source terrain, with for example the Amazon River and the Minjiang River having clays dominated by kaolinite^66,67^, while the Yangtze River and the Wadden sea estuary have clays dominated by illite and montmorillonite^9,10,68^. Although there is paucity of compositional data for rivers worldwide, an average clay composition in suspended sediments of 62% illite, 6% montmorillonite, and 11% kaolinite can be derived from the above literature. Based on our SCM results, it is predicted that around 54% (0.5 ppb), 8% (0.08 ppb), and 24% (0.2 ppb) of Cd can be released from montmorillonite, kaolinite, and illite surfaces, respectively, if we assume a modern river average of ca. 1 ppb of total Cd (dissolved as free ions plus adsorbed on suspended sediments – see Supplementary Table 1). Applying the average suspended clay composition and the smallest of the abovementioned fractions of clay in riverine suspended sediments (10%), the total discharge of clay into estuaries is 1.35 Gt. This translates into an annual contribution of 256.5 t of Cd to the oceans. Our results demonstrate the key role that clays play in transporting trace elements, including Cd, to the oceans, which suggests that clays supply a considerable fraction of the trace metals inventory to marine sediments.

## Supplementary information

Supplementary Information.
