# Supplementary material for: Clay minerals as a source of cadmium to estuaries
Source: Sci Rep. 2020 Jun 26;10:10417. doi: 10.1038/s41598-020-67279-w (PMC7320025; doi:10.1038/s41598-020-67279-w)
Supplement: Supplementary file 1 — Supplementary Information. [file 41598_2020_67279_MOESM1_ESM.docx]

**Clay minerals as a source of cadmium to estuaries**

Weiduo Hao^1*^, Teruhiko Kashiwabara^2^, Rong Jin^1^, Yoshio Takahashi^3^, Murray Gingras^1^, Daniel S. Alessi^1^, Kurt O. Konhauser^1^

^1^Department of Earth & Atmospheric Sciences, University of Alberta, Edmonton, Alberta, T6G 2E3, Canada.

^2^Japan Agency for Marine–Earth Science and Technology (JAMSTEC), 2-15, Natsushimacho, Yokosuka, Kanagawa 2370061, Japan

^3^Department of Earth and Planetary Science, The University of Tokyo, 7-3-1 Hongo, Bunkyo-ku, Tokyo, 1130033, Japan

*Corresponding author:

Weiduo Hao

3-11 Earth Sciences Building, University of Alberta, Edmonton, AB, Canada

T6G 2E3

587 990 2819

whao@ualberta.ca

Supplementary Table 1. A compilation of Cd concentrations in suspended sediments (μg/g).

|  | River | Estuary | Ocean |
| --- | --- | --- | --- |
| Huanghe | 0.39 (Ref ^48^) | 0.11 (Ref ^49^) | 0.02 (Ref ^49^) |
| Yangtze River | 4.73 (Ref ^50^) | 0.03 (Ref ^51^) | 0.09 (Ref ^52^) |
| Pearl River | 0.84 (Ref ^53^) | 0.46 (Ref ^54^) | 0.29 (Ref ^55^) |
| Mississippi River | 1.3 (Ref ^56^) | 1.5 (Ref ^56^) | 0.3 (Ref ^57^) |
| Seine River | 2.47 (Ref ^58^) | 6.18 (Ref ^58^) | 2.25 (Ref ^58^) |
| Neil River | 1.17 (Ref ^59^) | 0.23 (Ref ^60^) | 0.49 (Ref ^61^) |

Supplementary Table 2. The input parameters for FITEQL modelling of Cd adsorption onto the three clays.

| Log K of protonation reactions | | | ≡LH+Na^+^↔H^+^+≡LNa | | ≡XOH↔H^+^+≡XO^-^ | | ≡XOH+H^+^↔≡XOH_2_^+^ | |
| --- | --- | --- | --- | --- | --- | --- | --- | --- |
| Kaolinite (NEM) | Ka | IS=0.01M | -8.027 | | -7.274 | | 5.347 | |
|  |  | IS=0.56M | -8.973 | | -5.971 | | 4.437 | |
|  | Site density | IS=0.01M | 1.020E-04 | | 2.322E-05 | | | |
|  |  | IS=0.56M | 5.865E-05 | | 2.166E-05 | | | |
| Kaolinite (CCM) | Ka | IS=0.01M | -5.726 | | -9.271 | | 8.442 | |
|  |  | IS=0.56M | -5.247 | | -8.899 | | 7.902 | |
|  | Site density | IS=0.01M | 3.490E-05 | | 2.427E-05 | | | |
|  |  | IS=0.56M | 3.427E-05 | | 1.588E-05 | | | |
| Montmorillonite (NEM) | Ka | IS=0.01M | -3.657 | | -10.440 | | 9.996 | |
|  |  | IS=0.56M | -5.083 | | -9.917 | | 9.821 | |
|  | Site density | IS=0.01M | 9.286E-05 | | 2.147E-04 | | | |
|  |  | IS=0.56M | 4.844E-05 | | 1.448E-04 | | | |
| Montmorillonite (CCM) | Ka | IS=0.01M | -3.625 | | -10.353 | | 11.229 | |
|  |  | IS=0.56M | -5.046 | | -9.790 | | 10.679 | |
|  | Site density | IS=0.01M | 9.050E-05 | | 2.146E-04 | | | |
|  |  | IS=0.56M | 4.597E-05 | | 1.443E-04 | | | |
| Illite (NEM) | Ka | IS=0.01M | -3.934 | | -10.627 | | 9.643 | |
|  |  | IS=0.56M | -5.030 | | -10.406 | | 9.367 | |
|  | Site density | IS=0.01M | 5.243E-05 | | 8.310E-05 | | | |
|  |  | IS=0.56M | 4.343E-05 | | 6.235E-05 | | | |
| Illite (CCM) | Ka | IS=0.01M | -6.389 | | -10.243 | | 9.761 | |
|  |  | IS=0.56M | -5.517 | | -9.647 | | 9.475 | |
|  | Site density | IS=0.01M | 5.655E-05 | | 8.788E-05 | | | |
|  |  | IS=0.56M | 5.055E-05 | | 6.971E-05 | | | |
|  |  |  |  |  | |  | |  |
| Parameters used in CCM | Surface area (m^2^/g) | | Suspension density (g/L) | | Inner-layer capacitance (F/m^2^) | | | |
| kaolinite | 18.15 | | 1 | | 16.0 | | | |
| montmorillonite | 22.21 | | 1 | | 14 | | | |
| illite | 21.06 | | 1 | | 8.0 | | | |
|  |  |  |  |  | |  | |  |
| Log K of hydrolysis reactions and chloride complex | River | | | Marine | | | | |
| Cd^2+^+H_2_O↔CdOH^+^+H^+^ | -10.010 | | | -9.835 | | | | |
| Cd^2+^+2H_2_O↔Cd(OH)_2_ | -20.210 | | | -20.035 | | | | |
| Cd^2+^+3H_2_O↔Cd(OH)_3_^-^+3H^+^ | -31.700 | | | -31.700 | | | | |
| Cd^2+^+4H_2_O↔Cd(OH)_4_^2-^+4H^+^ | -47.480 | | | -47.830 | | | | |
| 2Cd^2+^+H_2_O↔Cd_2_(OH)_3_^+^+H^+^ | -9.490 | | | -9.665 | | | | |
| 4Cd^2+^+4H_2_O↔Cd_4_(OH)_4_^4+^+4H^+^ | -32.979 | | | -32.330 | | | | |
| Cd^2+^+Cl^-^↔CdCl^+^ | 2.18 | | | 2.53 | | | | |
| Cd^2+^+2Cl^-^↔CdCl_2_ | 2.97 | | | 3.49 | | | | |
| Cd^2+^+3Cl^-^↔CdCl_3_^-^ | 2.37 | | | 2.89 | | | | |

Note, the protonation and site density data are from Hao et al. (2019a); Cd hydrolysis and chloride complex data are from Baes and Mesmer (1997) and Liu et al. (2018).

Supplementary Table 3. Calculated binding constants for Cd adsorption onto three clay minerals.

|  |  | Freshwater | | | Marine | | |
| --- | --- | --- | --- | --- | --- | --- | --- |
|  |  | Log(K_LCd_) | Log(K_XOCd_) | V(Y) | Log(K_LCd_) | Log(K_XOCd_) | V(Y) |
| Kaolinite | NEM | -3.164 | -1.664 | 0.142 | -0.514 | 0.942 | 0.005 |
|  | CCM | -0.930 | -1.516 | 0.012 | 1.331 | -0.522 | 0.018 |
| Illite | NEM | -1.209 | 0.199 | 0.179 | 1.388 | -1.425 | 0.009 |
|  | CCM | -0.583 | -0.104 | 0.118 | 1.495 | -1.746 | 0.004 |
| Montmorillonite | NEM | -0.687 | -2.214 | 0.596 | 1.551 | -1.173 | 0.015 |
|  | CCM | 0.496 | -1.021 | 0.573 | 2.137 | -0.364 | 0.014 |

Supplementary Table 4. Maximum Cd adsorption onto three clay minerals at freshwater and marine conditions.

|  | Freshwater (mg/g) | Seawater (mg/g) |
| --- | --- | --- |
| Kaolinite | 2.31 | 1.00 |
| Illite | 3.59 | 1.94 |
| Montmorillonite | 36.76 | 4.73 |

Supplementary Table 5 Structural parameters of Cd adsorption onto three clay minerals at river and marine conditions.

|  | Shell | CN | R (Å) | dE (eV) | σ^2^ (Å^2^) | R factor (%) |
| --- | --- | --- | --- | --- | --- | --- |
| Kao river | Cd-O^*^ | 6.0 | 2.28 (0.03) | 3.3 | 0.007 | 0.84 |
| Illite river | Cd-O^*^ | 6.0 | 2.27 (0.03) | 2.9 | 0.009 | 1.39 |
| Mont river | Cd-O^*^ | 6.0 | 2.26 (0.03) | 1.3 | 0.006 | 0.89 |
| Kao marine | Cd-O^*^ | 6.0 | 2.28 (0.04) | 5.1 | 0.011 | 0.11 |
|  | Cd-Al | 2.2 | 3.16 (<0.01) | 5.1 | 0.010 |  |
| Illite marine | Cd-O^*^ | 6.0 | 2.29 (0.03) | 5.4 | 0.011 | 0.27 |
|  | Cd-Al | 1.5 | 3.36 (<0.01) | 5.4 | 0.009 |  |
| Mont marine | Cd-O^*^ | 6.0 | 2.26 (0.04) | 4.9 | 0.010 | 2.18 |
|  | Cd-Al | 2.1 | 3.16 (<0.01) | 4.9 | 0.009 |  |

^*^ CN is fixed to 6 for all of the Cd-O shells. The R factor is expressed as: R(%)=Ʃ[χ_obs_(E)- χ_cal_(E)]^2^/ Ʃ[χ_obs_(E)]^2^, where χ_obs_(E) and χ_cal_(E) are the experimental and calculated adsorption coefficients at a given energy (E), respectively. The estimated errors (standard deviations) are given in parentheses.

|  | Shell | CN | R (Å) | dE (eV) | σ^2^ (Å^2^) | R factor (%) |
| --- | --- | --- | --- | --- | --- | --- |
| Kao river | Cd-O^*^ | 6.0 (0.7) | 2.28 (0.03) | 3.3 | 0.007 | 0.84 |
| Illite river | Cd-O^*^ | 6.1 (0.7) | 2.27 (0.03) | 2.9 | 0.009 | 1.37 |
| Mont river | Cd-O^*^ | 6.1 (0.7) | 2.26 (0.03) | 1.3 | 0.006 | 0.86 |
| Kao marine | Cd-O^*^ | 6.0 (0.8) | 2.28 (0.04) | 5.1 | 0.011 | 0.11 |
|  | Cd-Al | 2.2 (0.1) | 3.16 (<0.01) | 5.1 | 0.010 |  |
| Illite marine | Cd-O^*^ | 6.0 (0.7) | 2.29 (0.03) | 5.4 | 0.011 | 0.28 |
|  | Cd-Al | 1.5 (0.1) | 3.36 (<0.01) | 5.4 | 0.009 |  |
| Mont marine | Cd-O^*^ | 6.0 (0.7) | 2.26 (0.05) | 4.9 | 0.010 | 2.19 |
|  | Cd-Al | 2.1 (0.1) | 3.16 (<0.01) | 4.9 | 0.009 |  |

^*^ σ^2^ is fixed for all of the Cd-O shells to float the CN. The R factor is expressed as: R(%)=Ʃ[χ_obs_(E)- χ_cal_(E)]^2^/ Ʃ[χ_obs_(E)]^2^, where χ_obs_(E) and χ_cal_(E) are the experimental and calculated adsorption coefficients at a given energy (E), respectively. The estimated errors (standard deviations) are given in parentheses.
